# Females and males respond differently to calls impaired by noise in a tree frog

**DOI:** 10.1002/ece3.7761

**Published:** 2021-06-06

**Authors:** Haodi Zhang, Bicheng Zhu, Ya Zhou, Qiaoling He, Xiaoqian Sun, Jichao Wang, Jianguo Cui

**Affiliations:** ^1^ CAS Key Laboratory of Mountain Ecological Restoration and Bioresource Utilization & Ecological Restoration and Biodiversity Conservation Key Laboratory of Sichuan Province Chengdu Institute of Biology Chinese Academy of Sciences Chengdu China; ^2^ University of Chinese Academy of Sciences Beijing China; ^3^ Ministry of Education Key Laboratory for Ecology of Tropical Islands, College of Life Sciences Hainan Normal University Haikou China

**Keywords:** acoustic communication, female choice, male–male competition, noise interference, signal recognition

## Abstract

Both human and nonhuman animals communicating acoustically face the problem of noise interference, especially anurans during mating activities. Previous studies concentrated on the effect of continuous noise on signal recognition, but it is still unknown whether different notes in advertisement calls impaired by noise affect female choice and male–male competition or not. In this study, we tested female preferences and male‐evoked vocal responses in serrate‐legged small tree frog (*Kurixalus odontotarsus*), by broadcasting the five‐note advertisement call and the advertisement call with the second, third, or fourth note replaced by noise, respectively. In phonotaxis experiments, females significantly discriminated against the advertisement call with the fourth note impaired by noise, although they did not discriminate against other two calls impaired by noise, which indicates that the negative effect of noise on female preference is related to the order of impaired notes in the advertisement call. In playback experiments, males increased the total number of notes in response to noise‐impaired calls compared with spontaneous calls. More interestingly, the vocal responses evoked by noise‐impaired calls were generally similar to those evoked by complete advertisement calls, suggesting that males may recognize the noise‐impaired calls as complete advertisement calls. Taken together, our study shows that different notes in advertisement calls replaced by noise have distinct effects on female choice and male–male competition.

## Graphical abstract

In this study, we tested female preference and male‐evoked vocal responses to the advertisement call and advertisement call with different note replaced by noise in a tree frog. We found that the effect of noise on female choice is related to the order of different replaced notes in the call, while males failed to distinguish between the advertisement call and the call with different notes replaced by noise.

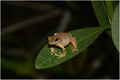

## 1 INTRODUCTION

Acoustic communication is essential for both survival and reproduction success of vocalizing animals (Fröhlich & Ciach, 2017; Wells & Schwartz, 2007). However, various environmental factors (e.g., noise) can interfere with signal detection and limit the distance at which a sound can be used to defend territories or attract mates (Lohr et al., 2003; Wiley, 2015; Wollerman, 1999). For most anurans, natural noise arising from conspecific and heterospecific calls is usually the major acoustic interference in breeding sites (Bee, 2007a, 2008a; Marshall et al., 2006; Wollerman & Wiley, 2002). Although most studies have explored the interference of continuous noise on signal recognition (Bee, 2007b, 2008a; Ward et al., 2013), few have investigated the impact of the noise impairing different notes in a call on both female choice and male–male competition (Song et al., 2020).

Since the environmental noise in a chorus usually changes dynamically in time and space (Coss et al., 2021; Lee et al., 2017), the components impaired by noise can be changed. Therefore, it is important to investigate the effect of calls with different notes impaired by environmental noise on signal recognition and sexual selection. Both female and male frogs rely on temporal characteristics of calls to evaluate potential mates and rivals (Gerhardt et al., 2000; Höbel, 2015; Marshall et al., 2006; Simmons, 2002), whether they can assess the duration of calls, in which different notes are impaired by noise, is crucial to their mating choice and male competition. Different components in calls may have distinct functions and biological significance (Fang et al., 2019; Yue et al., 2017). Previous studies revealed that the temporal order of components affects call recognition (Gerhardt et al., 2007; Wilczynski et al., 1999). Therefore, we hypothesize that the advertisement calls with different notes impaired by noise may affect female and male signal discrimination.

**FIGURE 1:**
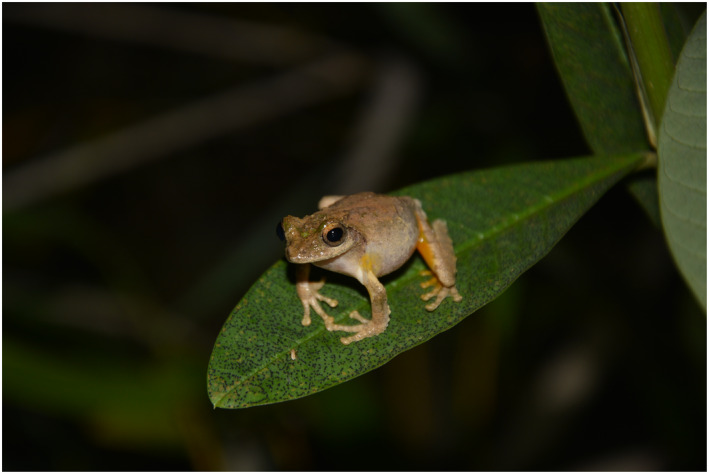
A male serrate‐legged small tree frog (*Kurixalus odontotarsus*)

The serrate‐legged small tree frog (*Kurixalus odontotarsus*) is an anuran species (Figure 1) which is widely distributed in the south of China, including Yunnan Province, Guizhou Province, Guangdong Province, Guangxi Province, and Hainan Province (Fei et al., 2010). Generally, males (always 5–8 members) aggregate spontaneously in breeding sites, calling to attract females and repel competitors. The natural chorus lasts for an average of 78.7 s (Zhu et al., 2021), in which there is a lot of noise from conspecific and heterospecific individuals. Male serrate‐legged small tree frogs always produce two kinds of notes: a wideband A note and a narrowband B note (Zhu et al., 2017). These notes make up three kinds of calls: advertisement calls, consisting of a series of A notes; aggressive calls, consisting of a series of B notes; and compound calls, consisting of a series of A notes and B notes. Our previous study demonstrated that male advertisement calls can attract females and elicit vocal responses of other males as well (Zhu, Wang, Zhao, et al., 2017). Therefore, the vocal characteristic and the complex acoustic environment make *K. odontotarsus* a suitable species for investigating the effects of the advertisement call with different notes impaired by noise on female choice and male–male competition.

In this study, we used the serrate‐legged small tree frog *K. odontotarsus* to investigate: (a) whether females discriminate against advertisement calls with partial notes impaired by noise; (b) whether the order of different impaired note in the call affects female signal discrimination; and (c) whether males respond differently to advertisement calls with different notes impaired by noise, through female phonotaxis tests and male‐evoked vocal response experiments.

## 2 MATERIALS AND METHODS

### 2.1 Study sites and subjects

Our study was conducted from May to August 2020 at the Mt. Diaoluo National Reserve in Hainan, China (18.44°N, 109.52°E, elevation of 933 m a.s.l.). The experiments were conducted from 20:00 to 01:00 the next day. The average temperature and relative humidity were 22.5 ± 0.8℃ and 97.6 ± 3.6%, respectively.

Gravid females and calling males were captured from the breeding sites per night. All subjects were housed individually in plastic cages (*L* × *W* × *H*: 28 × 18 × 15 cm) containing water and plants with natural temperature and photoperiod. The interval between collecting and testing was an average of 1 hr. Female phonotaxis experiments were conducted in a darkened sound‐attenuating chamber (*L* × *W* × *H*: 280 × 180 × 185 cm). Male playback experiments were carried out in enclosure constructed of wire mesh (*L* × *W* × *H*: 42 × 32 × 90 cm), which were far from choruses (Deng et al., 2020). Males could move freely in enclosure, with soil and plants provided.

To avoid recapturing in the wild on subsequent nights, passive integrated transponders tags (PIT, 60 mg; HT950, Guangzhou Hongteng Barcode Technology Co. Ltd.) were injected subcutaneously into the dorsum of frogs after testing, and the specific tag number was obtained by PIT scanner (HT8000; Guangzhou Hongteng Barcode Technology Co. Ltd.) (Pyke, 2005; Salazar et al., 2016). After the injection, the wound was wiped with diluted alcohol to avoid infection. All frogs used in our study were released to the captured site immediately after testing, after being held in the cages for an average of 3 hr between the capture and the end of the experimental procedures. Our subsequent observations showed that such treatment did not affect the activities of frogs in the wild, such as climbing and mating.

All applicable international, national, and/or institutional guidelines for the care and use of animals were followed. All procedures performed in studies involving animals were in accordance with the ethical standards of the Animal Care and Use Committee of Chengdu Institute of Biology, CAS (CIB2016008).

### 2.2 Acoustic stimuli

**FIGURE 2:**
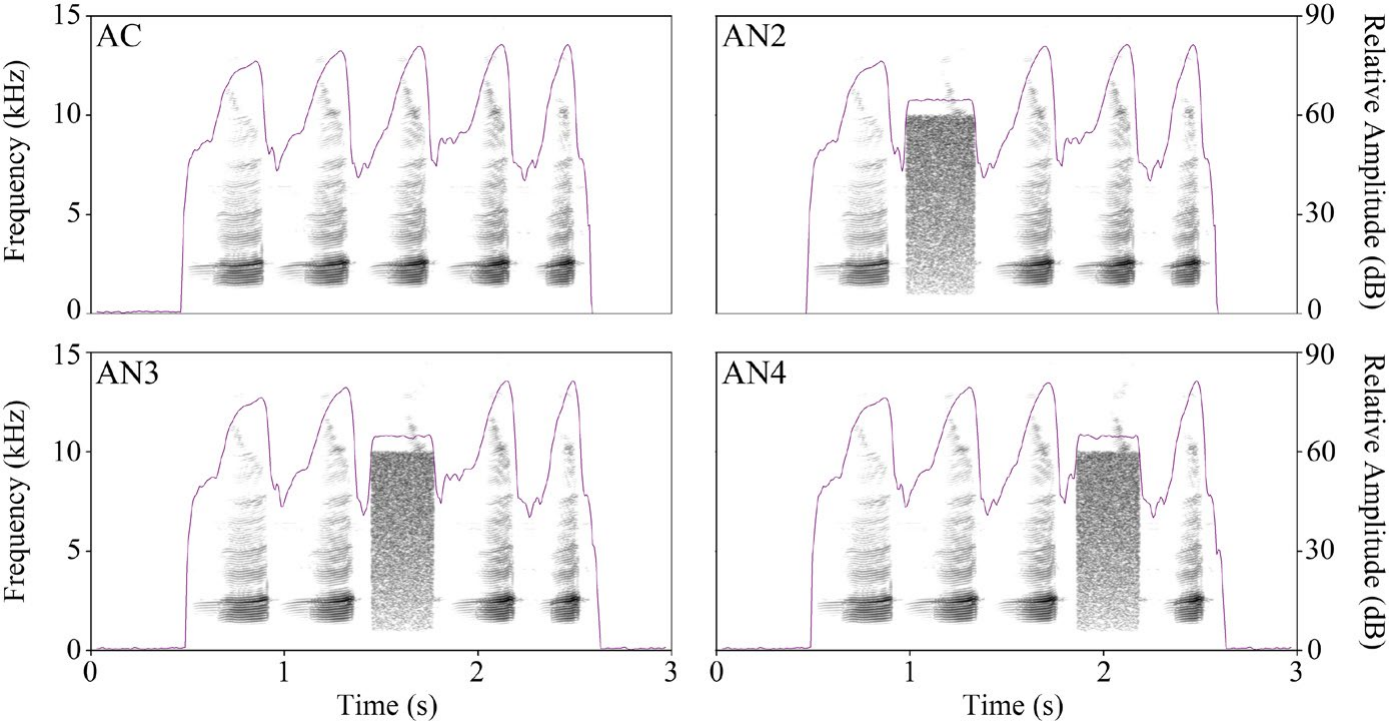
Spectrograms and relative amplitude of each acoustic stimulus. “AC,” five‐note advertisement call; “AN2,” advertisement call with the second note replaced by noise; “AN3,” advertisement call with the third note replaced by noise; “AN4,” advertisement call with the fourth note replaced by noise. The grayscale spectrogram indicates the frequency components of each stimulus. The purple line indicates the relative amplitude of each stimulus

The five‐note advertisement call (abbreviated as “AC”) is common in natural recordings, with an average duration of 2 s (Zhu, Wang, Brauth, et al., 2017). Our experiments shared several stimulus types in common, including the five‐note advertisement call (“AC”) and the advertisement call with the second (“AN2”), third (“AN3”) or fourth note (“AN4”) replaced by white noise (Figure 2). The frequency of experimental noise ranged from 1.0 to 10.0 kHz, which spanned all sensitive hearing range of *K. odontotarsus* (Zhu, Wang, Brauth, et al., 2017). The relative amplitude of white noise was −10.0 dB to the fifth note in each call, and the mean durations were 251.8 ± 15.3 ms, 242.7 ± 17.6 ms, and 240.5 ± 14.6 ms, respectively. In accordance with the work of Ringler et al. (2017), we used the “Inband Power” function in the software Raven Pro 1.6 (Bioacoustics Research Program 2011) to calculate the SNR of each noise‐impaired call. Our results showed that the SNR of each noise‐impaired call was very close, −5.87 ± 0.82 dB. All stimuli used in the experiments were constructed with natural calls by Adobe Audition 3.0 software (Adobe Systems Inc.; 44.1 kHz, 16 bits) and have equivalent call duration, 2 s on average. To minimize the effect of pseudoreplication, six advertisement calls derived from six different calling males were used to generate stimuli. These six natural calls from different individuals may reflect the call properties of the mean population values. When each female or male frog participated in the experiment, one of the six stimulus pairs from different males was randomly selected.

### 2.3 Female phonotaxis experiments

To investigate whether advertisement calls with different notes replaced by noise affect female preference, we performed 3 two‐stimulus choice tests. In all tests, one stimulus was a complete advertisement call (AC), another stimulus was a noise‐impaired call (AN2, AN3, or AN4). The three stimulus pairs were played randomly, and the two stimuli in each stimulus pair were presented alternately by two portable field speakers (SME‐AFS, Saul Mineroff Electronics), placed equidistant on the opposite side of the sound‐attenuating chamber, 220 cm apart. To control potential bias, stimulus pairs were randomly broadcast at peak level of 80 dB SPL (re 20 µPa, Z‐weighted), measured by a sound pressure level meter (AWA 6291, Hangzhou Aihua Instruments Co.) at the site where the female was released, 1 m away from the speaker.

Before each test, the subject was placed in the center of the chamber, under a transparent hemisphere with some holes at the bottom (12.8 cm in diameter, 1.3 mm thick) for 60 s to acclimate. After acclimation, the female frog was released to make a choice. For each test, we recorded the starting time (defined as the moment the hemisphere was raised), the time female frog leaving the release point as the latency to move, and the time female frog arriving the choice zone (i.e., within 10 cm of a speaker) as the latency to choose. The latency to move and the latency to choose can indicate whether females showed different levels of motivation for identifying diverse stimulus pairs. Furthermore, we scored a choice when the female approached the choice zone within 10 min without simply following the wall. If she did not reach the choice zone within 10 min, or was unable to leave the release location in 5 min, we scored no choice. All behaviors of females were observed on a monitor using a wide‐angle lens video system with an infrared light source (also see Zhu, Wang, Zhao, et al., 2017).

Each female was tested only once with any given stimulus. To avoid experimental fatigue, each subject was allowed a 3‐min break between consecutive tests. During the interval of each test, we mopped the floor to keep the arena moist and to eliminate possible chemical cues. After the experiments, female body size was measured (snout–vent length: 47.34 ± 2.37 mm, *N* = 52; body mass: 7.17 ± 1.10 g, *N* = 52).

### 2.4 Male playback experiments

To investigate whether advertisement calls with different notes replaced by noise affect male competition, we carried out male‐evoked vocal response experiments. The acoustic stimuli (AC, AN2, AN3, and AN4) were broadcast in a random order using a speaker (SME‐AFS, Saul Mineroff Electronics) at 1 m away from the enclosure. Prior to testing each subject, each stimulus was calibrated to 80 dB SPL (re 20 µPa, Z‐weighted) using a sound level meter (AWA 6,291; Hangzhou Aihua Instruments Co.). We recorded spontaneous calls for 3 min in a quiet environment (abbreviated as “S”) using a sound recorder (R6822; Aigo Digital Technology Co. Ltd.). Then, we randomly played back the stimuli (AC, AN2, AN3, AN4) for 3 min and recorded male‐evoked vocal responses during each playback period. The interval between each stimulus was 3 min. We analyzed the vocalization parameters of spontaneous calls and evoked calls during each playback period. Males who failed to vocalize spontaneously were excluded from our experiments. After experiments, males were measured (snout–vent length: 33.58 ± 1.35 mm, *N* = 41; body mass: 2.03 ± 0.21 g, *N* = 41).

### 2.5 Analysis and statistics

The spectrogram and relative amplitude of acoustic stimuli were visualized using free PRAAT software (Boersma, 2002). We used Adobe Audition 3.0 software (Adobe Systems Inc.) to count nine parameters of male vocalizations: the total number of calls; total number of notes (representing the overall vocal responses); number of advertisement calls; number of A notes; maximum number of A notes (representing the intensity of advertisement calls); number of aggressive calls; number of B notes; maximum number of B notes (representing the intensity of aggressive calls); and number of compound calls (representing the complexity of calls). Female latency and nine parameters of male‐evoked vocal responses were analyzed using SPSS 21.0 software (SPSS Inc.) and visualized using Origin 2017 software (OriginLab Corp.). The two‐tailed binomial test was used to evaluate the proportion of females who chose noise‐impaired calls. The generalized linear mixed model (GLMM) was utilized to analyze female latency and male‐evoked vocal responses (Bolker et al., 2009). We first explored various null models to find the random structure that best fitted our data based on the Akaike information criterion. Then, we created model with a Poisson error structure and log‐link function. Specifically, we set the acoustic environment (female latency: AC and AN2, AC and AN3, AC and AN4; male‐evoked vocal responses: S, AC, AN2, AN3, AN4) as a fixed effect and FrogID as a random effect. We chose pairwise comparisons with the estimated marginal means contrasts to complete multiple comparisons (adjust for multiple comparisons using least significant difference). All data were expressed as the mean ± *SD*, and *p* < 0.05 was considered to be statistically significant.

## 3 RESULTS

### 3.1 Female phonotaxis experiments

**FIGURE 3:**
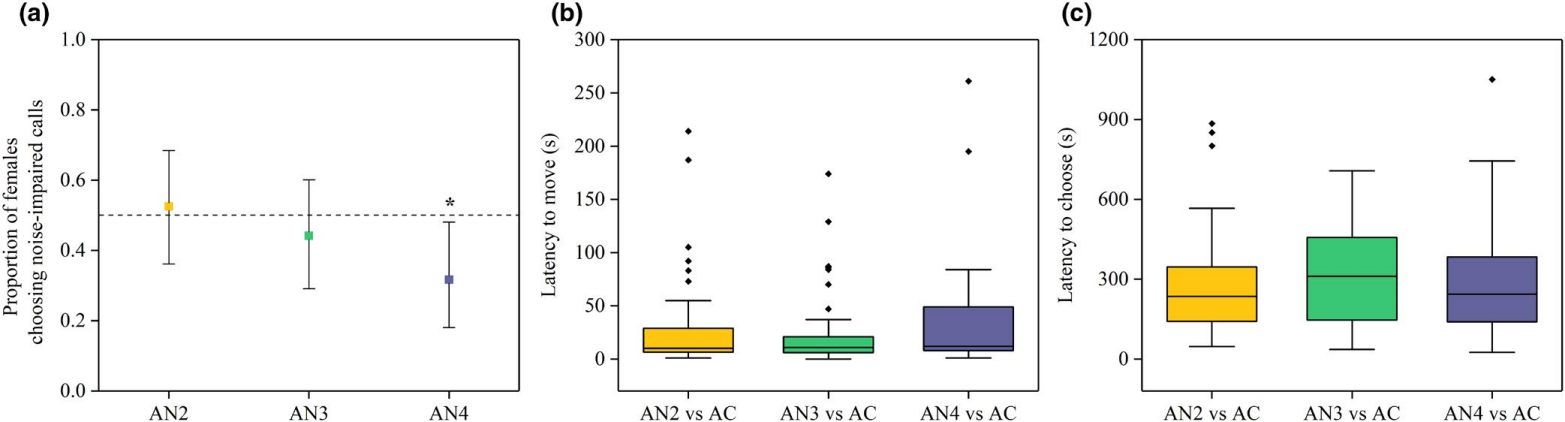
Female preferences and motivations in phonotaxis experiments. (a) Proportion of females choosing noise‐impaired calls. (b) The latency to move and (c) the latency to choose under different stimulus pairs. Each stimulus pairs consists of a five‐note advertisement call (AC) and a noise‐impaired call (AN2, AN3, or AN4). The median values of the latency to move were 10, 11, and 12 s, the latency to choose were 235.5, 311 and 244 s, respectively. In (a), two‐tailed binomial test; *p < 0.05. In (b) and (c), generalized linear mixed model (GLMM)

In female phonotaxis experiments, we tested female preferences to different noise‐impaired calls. For AN2 and AC comparison, 50 females were utilized and 10 subjects failed to make a choice (failure rate: 20.0%), 52.5% of females chose AN2 over AC (21 vs. 19; two‐tailed binomial test: *p* = 0.875, *N* = 40). For AN3 and AC comparison, 50 females were utilized and 7 subjects failed to make a choice (14.0%), 44.2% of females chose AN3 over AC (19 vs. 24; two‐tailed binomial test: *p* = 0.542, *N* = 43). Lastly, 52 females were utilized and 11 subjects failed to make a choice in AN4 and AC comparison (21.2%), 31.7% of females chose AN4 over AC (13 vs. 28; two‐tailed binomial test: *p* = 0.028, *N* = 41; Figure 3a).

We found that there was no significant difference in the latency to move (AN2 vs. AC: 30.4 ± 47.0 s; AN3 vs. AC: 24.9 ± 36.0 s; AN4 vs. AC: 33.8 ± 50.0 s; GLMM: *F*_(2,121)_ = 0.293, *p* = 0.747; Figure 3b), as well as the latency to choose (AN2 vs. AC: 283.2 ± 205.2 s; AN3 vs. AC: 305.0 ± 179.2 s; AN4 vs. AC: 297.9 ± 214.0 s; GLMM: *F*_(2,121)_ = 0.176, *p* = 0.838; Figure 3c). Thus, the difference in the proportions of females who chose noise‐impaired calls was not caused by motivation.

### 3.2 Male playback experiments

**FIGURE 4:**
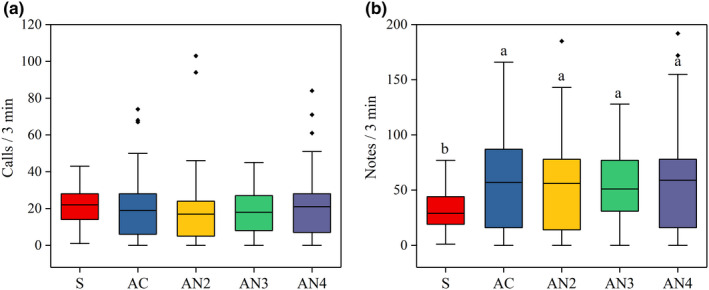
The overall vocal responses in male playback experiments. (a) The total number of calls, (b) total number of notes during the spontaneous period (S) and playback periods (AC, AN2, AN3, and AN4). The median values of the total number of calls were 22, 19, 18, 18, and 21, total number of notes were 29, 57, 56, 51, and 59 respectively. GLMM; different superscript letters indicate significant differences among different treatments (*p* < 0.05)

In male playback experiments, we measured the call parameters of 41 males at each period. Although there was no significant difference in total number of calls between the spontaneous period and each playback period (GLMM: *F*_(4,200)_ = 0.871, *p* = 0.482; Figure 4a), males produced significantly more total number of notes (GLMM: *F*_(4,200)_ = 4.957, *p* ≤ 0.001; Figure 4b) during playback periods compared with the spontaneous period. There was no difference in the overall vocal responses of males to the complete advertisement call and each noise‐impaired call (Table 1; Figure 4).

**TABLE 1:** The results of male‐evoked vocal responses

	Stimulus				
	S	AC	AN2	AN3	AN4
total calls	20.7 ± 9.9	20.6 ± 18.1	20.2 ± 21.0	18.2 ± 11.2	22.1 ± 18.7
total notes	32.7 ± 20.1	59.9 ± 48.0	53.7 ± 43.4	54.1 ± 32.3	60.5 ± 47.2
advertisement calls	20.0 ± 10.4	10.8 ± 8.7	11.6 ± 10.1	12.4 ± 8.7	13.0 ± 9.2
A notes	30.8 ± 19.5	33.7 ± 28.3	34.6 ± 29.7	37.9 ± 27.9	37.5 ± 26.8
max A notes	2.5 ± 1.6	3.7 ± 2.6	3.9 ± 2.7	4.4 ± 2.0	3.8 ± 2.3
aggressive calls	0.7 ± 1.2	9.2 ± 13.3	8.4 ± 16.3	5.5 ± 7.1	8.6 ± 15.0
B notes	1.9 ± 4.7	26.1 ± 35.0	19.1 ± 28.5	16.2 ± 17.1	23.0 ± 38.2
max B notes	1.3 ± 2.7	6.1 ± 5.8	3.7 ± 3.9	5.9 ± 5.5	5.5 ± 6.0

Note

S, spontaneous call; AC, five‐note advertisement call; AN2, advertisement call with the second note replaced by noise; AN3, advertisement call with the third note replaced by noise; AN4, advertisement call with the fourth note replaced by noise.

**FIGURE 5:**
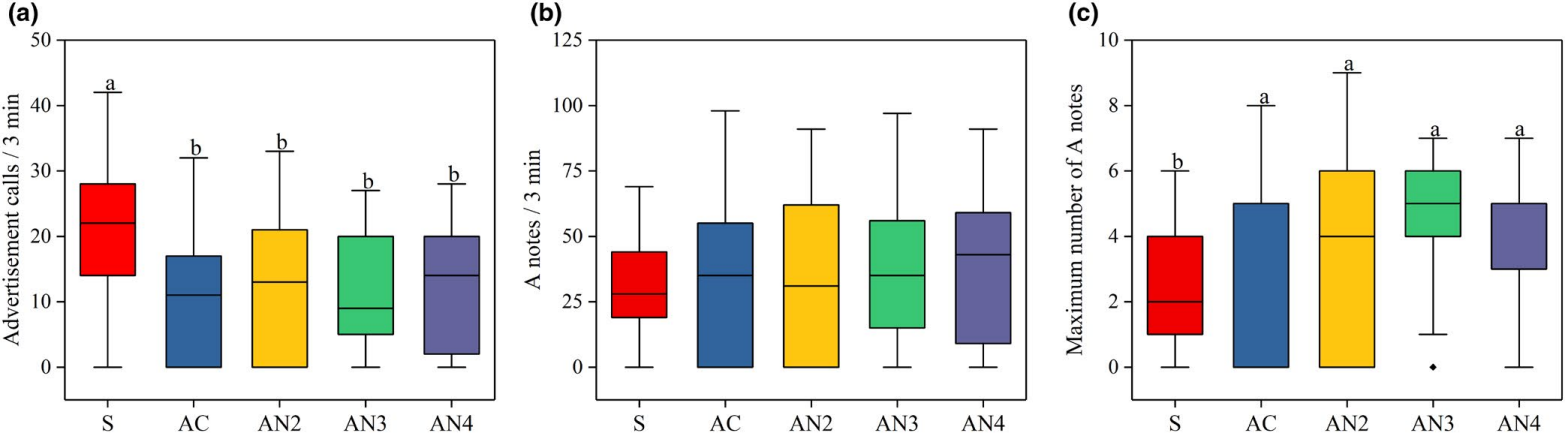
The intensity of advertisement calls in male playback experiments. (a) The number of advertisement calls, (b) number of A notes, (c) maximum number of A notes during the spontaneous period (S) and playback periods (AC, AN2, AN3, and AN4). The median values of the number of advertisement calls were 22, 11, 13, 9, and 14, number of A notes were 28, 35, 31, 35, and 43, and maximum number of A notes were 2, 5, 4, 5, and 5 respectively. GLMM; different superscript letters indicate significant differences among different treatments (*p* < 0.05)

In terms of the intensity of advertisement calls, males produced significantly less advertisement calls in response to playback periods than the spontaneous period (GLMM: *F*_(4,200)_ = 4.018, *p* = 0.004; Figure 5a). In addition, although there was no significant difference in the number of A notes between the spontaneous period and each playback period (GLMM: *F*_(4,200)_ = 0.585, *p* = 0.674; Figure 5b), males in playback periods produced significantly more maximum number of A notes (GLMM: *F*_(4,200)_ = 3.511, *p* = 0.009; Figure 5c) than in the spontaneous period. In general, there was no difference in the intensity of advertisement calls between the complete call and each noise‐impaired call (Table 1; Figure 5).

**FIGURE 6:**
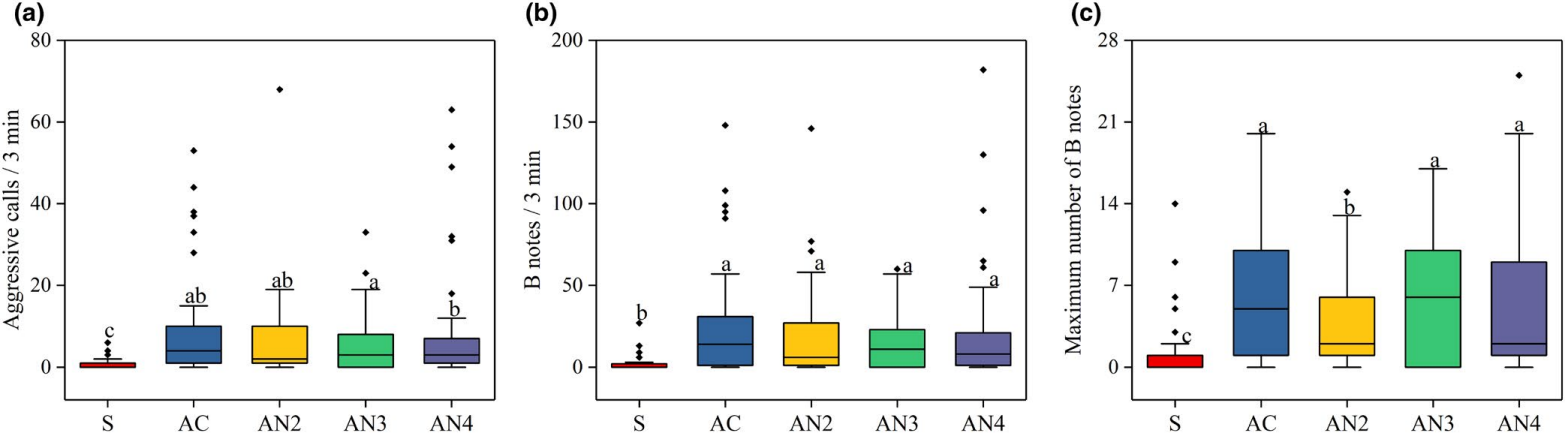
The intensity of aggressive calls in male playback experiments. (a) The number of aggressive calls, (b) number of B notes, (c) maximum number of B notes during the spontaneous period (S) and playback periods (AC, AN2, AN3, and AN4). The median values of the number of aggressive calls were 0, 4, 2, 3, and 3, and number of B notes were 0, 14, 6, 11, and 8, maximum number of B notes were 0, 5, 2, 6, and 2, respectively. GLMM; different superscript letters indicate significant differences among different treatments (*p* < 0.05)

In terms of the intensity of aggressive calls, males produced more aggressive calls in playback periods than in the spontaneous period (GLMM: *F*_(4,200)_ = 38.157, *p* ≤ 0.001; Figure 6a). Meanwhile, compared with the spontaneous period, the number of B notes (GLMM: *F*_(4,200)_ = 20.399, *p* ≤ 0.001; Figure 6b) and the maximum number of B notes (GLMM: *F*_(4,200)_ = 12.731, *p* ≤ 0.001; Figure 6c) were greatly increased in playback periods. Although males produced a different number of aggressive calls and maximum number of B notes in playback periods, the difference in the aggressiveness of vocal responses between the complete call and each noise‐impaired call was slight (Table 1; Figure 6).

As for call complexity, compound calls were only generated during playback periods, not the spontaneous period. Seventeen males (41.5%) emitted 73 compound calls which consisted of 3.41 ± 1.32 (mean ± *SD*) A notes and 3.04 ± 2.90 (mean ± *SD*) B notes. Among them, 26 compound calls were produced during AC, 11 compound calls were produced during AN2, 15 compound calls were produced during AN3, and 21 compound calls were produced during AN4.

## 4 DISCUSSION

Our study investigated the behavioral responses of female and male *K. odontotarsus* to advertisement calls with different notes replaced by noise. We found that females discriminated against the advertisement call with the fourth note impaired by noise, but do not discriminate against other two noise‐impaired calls, which were not affected by female motivation to different stimulus pairs. Meanwhile, compared with the spontaneous period, males increased vocal responses during noise‐impaired calls were played back. However, the vocal responses evoked by each noise‐impaired call were generally similar to those evoked by the complete advertisement calls.

Previous studies have shown that whether the impairment of call structures has a negative effect on female choice varies among species: female *Hyla versicolor* make more recognition errors when conspecific call structures are overlapped by heterospecific calls (Marshall et al., 2006), while female *Hyla chrysoscelis* and *Hyla cinerea* can accurately respond to conspecific calls without being negatively affected by heterospecific call interference (Höbel, 2015; Marshall et al., 2006). Our results demonstrated that the discrimination of female *K. odontotarsus* against noise‐impaired calls is related to the order of the impaired note in the advertisement call.

Since many animals respond differently to distinct components of the same vocalizations at both behavioral and neural levels (Fang et al., 2019; Yu et al., 2011; Yue et al., 2017), it is reasonable to speculate that different notes of advertisement calls contribute differently to mate selection of female serrate‐legged small tree frogs. This may be a reason for female different responses to the advertisement calls with different notes replaced by noise. Moreover, a class of temporally selective neurons (interval‐counting neurons), identified in the torus semicircularis of several anuran species (Edwards et al., 2002), may play a significant role in signal recognition. The interval‐counting neurons that were thought to be related to the effect of gap location on call recognition in gray tree frogs (Henderson & Gerhardt, 2013) may also be related to the effect of noise‐impaired location on call recognition in serrate‐legged small tree frogs.

Temporal characteristics of calls are critical for signal recognition and mate choice (Höbel, 2015; Marshall et al., 2006; Wilczynski et al., 1999). For example, call duration is a reliable indicator of male heritable genetic quality (Welch et al., 1998). However, environmental noise arising from other individuals in leks can impair the assessment of call duration (Schwartz, 1987). Previous studies in *H. versicolor* showed that female preferences for longer calls are weakened or reversed in chorus noise compared to quiet conditions (Bee, 2008b). Different from this result, we found that female serrate‐legged small tree frogs would consider the temporal order of notes that are impaired by noise in the advertisement call to assess the call duration. Our result provides a potential perspective for understanding the process of call duration assessment in noisy environment.

In addition, for some vocalizing taxa, the receiver's auditory system can actively fill the part that is masked by noise to create a complete auditory object, resulting in an auditory induction (Miller et al., 2001; Petkov et al., 2003; Seeba & Klump, 2009). Auditory induction has been proved to play an important role in promoting call recognition under noise interference. Female *K. odontotarsus* discriminated against the five‐note advertisement call with the second note missing (39 vs. 68, B. C. Zhu, unpublished data), but they did not discriminate against the advertisement call with the second note replaced by noise (present study), which suggests that female serrate‐legged small tree frog may experience auditory induction. This may be of great significance for revealing how frogs overcome noise masking in the chorus.

Previous studies have shown that the presence or absence of noise affects male vocal responses (Penna & Hamilton‐West, 2007; Penna et al., 2005). Surprisingly, our study showed that male calling responses evoked by different noise‐impaired calls are generally similar to those evoked by complete advertisement calls. These findings are consistent with the study of *Rhacophorus zhoukaiyae* (Song et al., 2020), that is, males recognize the call of individual notes impaired by noise as a complete call. Although the total number of calls and total number of notes are the same when three types of noise‐impaired calls are played back, the number of compound calls, the number of aggressive calls, and maximum number of B notes produced by males are different, suggesting that these three types of noise‐impaired calls have slight differences in the process of male recognition.

In fact, in a chorus, not only do females use calls to evaluate the quality of potential mates, but males also use their vocalizations to assess competitors. Our results indicate that males may ignore the middle part of calls when evaluating competitors in a chorus. One possible explanation is that males may experience auditory induction when individual note in an advertisement call is replaced by noise (Bregman, 1990; Miller et al., 2001). In general, our results provide a potential implication for understanding how males evaluate rivals in noisy environments (e.g., chorus).

Comparing the results of female phonotaxis experiments and male playback experiments, we found that the noise‐impaired calls have different effects on female choice and male–male competition. According to previous studies, there are sexual differences in behavioral responses to the same acoustic signals in other species. For example, male túngara frogs (*Physalaemus pustulosus*) and green tree frogs (*H*. *cinerea*) are more likely to respond to heterospecific calls than conspecific females (Bernal et al., 2007; Höbel, 2015). The differences in behavioral responses between the sexes may be due to different methods of recognizing signals or different structures of auditory organs (Shen et al., 2011; Zhu, Wang, Zhao, et al., 2017). Females generally spend a longer time evaluating and localizing mates, which allows them to sample the signal multiple times, while males usually respond quickly to a rival's signal in a shorter time (Höbel, 2015). Therefore, our results suggest that females and males may have different ways of identifying the noise‐impaired calls, and sexual difference should be considered when studying the effect of noise on acoustic communication.

## CONFLICT OF INTEREST

The authors declare no competing interests.

## AUTHOR CONTRIBUTIONS

**Haodi Zhang:** Conceptualization (equal); Data curation (equal); Investigation (equal); Writing‐original draft (equal); Writing‐review & editing (equal). **Bicheng Zhu:** Conceptualization (equal); Data curation (equal); Investigation (equal); Writing‐original draft (equal); Writing‐review & editing (equal). **Ya Zhou:** Data curation (equal); Investigation (equal). **Qiaoling He:** Data curation (equal); Investigation (equal). **Xiaoqian Sun:** Data curation (equal); Investigation (equal). **Jichao Wang:** Investigation (equal); Resources (equal). **Jianguo Cui:** Conceptualization (equal); Funding acquisition (lead); Project administration (lead); Resources (equal); Writing‐original draft (equal); Writing‐review & editing (equal).

## Data Availability

Data used to generate the results and figures are available from the Dryad Digital Repository: https://doi.org/10.5061/dryad.wpzgmsbmf.
